
JOURNAL INFORMATION
==============================
NLM Title Abbreviation: Behav Neurol
Journal ID: Behav Neurol
NLM Journal ID: 8914585
Journal ID: BN

Behavioural Neurology

ISSN: 0953-4180
EISSN: 1875-8584
Publisher: Wiley

ARTICLE INFORMATION
==============================
PMCID: PMC5434330
PMID: 21422548
DOI: 10.3233/BEN-2010-0288
Article ID: 527414
Article version: 1
Subjects: Research Article

Working Memory in Alzheimer’s Disease and Frontotemporal Dementia

Stopford C. L. *
Thompson J. C.
Richardson A. M. T.
Neary D.
Snowden J. S.
Greater Manchester Centre for Neurosciences Salford UK
*C. L. Stopford: cheryl.stopford@manchester.ac.uk
Print publication date: 2010
Electronic publication date: 2011 Mar 7
Volume: 23
Issue: 4
First page: 177
Last page: 179
Received 2011 Jan 31; Accepted 2011 Jan 31
Copyright: Copyright © 2010 Hindawi Publishing Corporation and the authors.
Copyright year: 2010
License: This is an open access article distributed under the Creative Commons Attribution License, which permits unrestricted use, distribution, and reproduction in any medium, provided the original work is properly cited.
License URL: https://creativecommons.org/licenses/by/3.0/

==============================
Behavioural Neurology 23 (2010) 177–179
DOI 10.3233/BEN-2010-0288
IOS Press

### Working memory in Alzheimer's disease and frontotemporal dementia

C.L. Stopford*, J.C. Thompson, A.M.T. Richardson, D. Neary and J.S. Snowden
*Greater Manchester Centre for Neurosciences, Salford, UK*

### 1. Background

Working memory deficits are a recognised feature of Alzheimer's disease (AD) and can be dissociated from episodic memory impairment, with distinct patterns of breakdown in individuals [1]. Distinctions in clinical profile may have neurobiological relevance. We have previously shown [2] that patients presenting with a dense yet relatively circumscribed classical amnesia ("amnestic-AD", accounting for 10% in a series of 523 patients) were older and more likely to carry apolipoprotein $\varepsilon 4$ alleles than those presenting with a constellation of working memory, language and perceptuospatial deficits (labelled "typical- AD" on the basis that they accounted for 61% of cases). Such findings highlight the importance of careful characterisation of individual patients in experimental research. A goal of the present study was to understand the working memory deficit in AD whilst recognising these important distinctions. Existing research focuses on difficulties on dual-task paradigms, encouraging the notion of central executive dysfunction, and promoting a relationship between working memory deficits and frontal lobe pathology in AD [3]. Failures on standard tests of attention and executive function reinforce this interpretation. However, characteristic neuroimaging changes in early onset AD are in posterior hemispheres rather than frontal lobes. Moreover, 'frontal' behavioural characteristics are absent. Characteristic qualities of persistence, motivation, and concern for accuracy contrast markedly with the economy of effort and lack of engagement demonstrated by patients with frontotemporal dementia (FTD), the prototypical neurodegenerative disease of the frontal lobes. The presence of qualitative differences in cognitive profile [4] suggests that there are distinct contributions to performance failure. We took the novel approach of exploring the frontal dysexecutive contribution to working memory by comparing test performance in patients with AD and FTD. If the basis of the impairment is primarily 'frontal dysexecutive', then one would expect similar profiles in the two groups. However given the 'posterior' abnormalities shown on AD patients' imaging and absence of 'frontal' behavioural signs, we predicted that there ought to be qualitative differences on tests of working memory, attention, and executive function.

*Corresponding author. E-mail: Cheryl.Stopford@manchester.ac.uk.

### 2. Participants

In line with our earlier work [2], the main focus were "typical" patients ($n = 20$; age 52–68) presenting with a constellation of cortical symptoms. Those with a circumscribed "amnesic" presentation ($n = 18$; age 59–83) were included as a reference group. AD patients were classified based on clinical history of symptoms obtained at initial diagnostic assessment and performance on a locally developed neuropsychological screening assessment. There were no significant differences between the AD and FTD patients ($n = 26$; age 52–76) with regard to illness duration (4–6 years) or Clinical Dementia Rating scale score (CDR 1: mild). Clinical neuroimaging showed characteristic temporoparietal change in typical-AD, medial temporal changes in amnesic-AD, and frontal lobe abnormalities in FTD.

### 3. Methods

Tasks were administered to examine working memory (a modified Brown-Peterson test involving memory for three words with and without delay/distraction;

ISSN 0953-4180/10/$27.50 © 2010 – IOS Press and the authors. All rights reserved

Table 1

| Task | Measure | Result | Comment |
|---|---|---|---|
| **Working memory** | | | |
| Modified working memory test | Overall accuracy | Typ-AD $<$ FTD $<$ Amn-AD = control | Poorer performance in Typ-AD than other groups |
| | No distraction, no delay | Typ-AD $<$ FTD = Amn-AD = control | Typ-AD impaired even without distraction or delay |
| | With delay | Typ-AD $<$ FTD = Amn-AD = control | FTD impaired only when distraction present |
| | With distraction | Typ-AD $<$ FTD $<$ Amn-AD = control | Amn-AD performance normal |
| Digit span | Forward and reverse | Typ-AD = FTD $<$ Amn-AD = control | Span impaired in Typ-AD and FTD but not Amn-AD |
| | Sequencing Errors | FTD $>$ Typ-AD | More sequencing errors in FTD |
| Body part pointing | Accuracy: 4 body parts | Typ-AD $<$ FTD $<$ Amn-AD = control | Poorer performance in Typ-AD than other groups |
| | Omission errors | Typ-AD $>$ FTD | Typ-AD more prone to omission error ("forgetting" items), |
| | Sequencing errors | FTD $>$ Typ-AD (trend) | FTD show tendency to misorder items |
| Phonological similarity task | Overall letter span | Typ-AD = FTD $<$ Amn-AD = control | Reduced letter span in both Typ-AD and FTD |
| | Phonological Similarity effect | Absent in Typ-AD; Present in others | Normal phonological similarity effect in FTD but not Typ-AD. |
| Word length | Overall word span | Typ-AD $<$ FTD = Amn-AD = control | Reduced word span in Typ-AD only |
| | Word length effect | Present in all groups | Span affected by word length in all groups |
| Visual patterns | Visual span | Typ-AD = FTD $<$ Amn-AD = control | Reduced visual span in both Typ-AD and FTD |
| **Attention** | | | |
| Vigilance A: sustained attention | Detection accuracy | FTD $<$ Typ-AD = Amn-AD = control | FTD impaired accuracy, Typ-AD perform normally |
| | Presentation speed effect | More errors with faster presentation speed in patient groups, not controls | Accuracy in all patient groups affected by presentation speed |
| | Reaction time | Typ-AD = FTD = Amn-AD = control | Speed of response normal in all groups |
| Vigilance B: continuous performance | Overall accuracy | Typ-AD = FTD $<$ Amn-AD = control | Typ-AD and FTD both show impaired accuracy |
| | Reaction time | Typ-AD = FTD = Amn-AD = control | Response times normal in all groups |
| | Loss of track of task | Typ-AD present; FTD absent | Behavioural observations show loss of track in Typ-AD and distractibility in FTD |
| | Distractible behaviour | FTD present; Typ-AD absent | |
| **Executive Function** | | | |
| Go-No-Go | Inhibition (no-go) errors | FTD $>$ other groups | FTD make most 'no-go' responses and show marked effect of 'Go' to 'No-go' ratio |
| | Effect of Go:No-go ratio | FTD $>$ other groups | Both Typ-AD and FTD omit targets |
| | Omission (go) errors | Typ-AD = FTD $<$ Amn-AD = control | Amn-AD perform normally |
| Set switching | Accuracy | FTD $<$ Typ-AD $<$ Amn-AD $<$ control | Impaired set switching in all patient groups, worst in FTD |
| | Response time | Typ-AD $<$ Amn-AD $<$ FTD = control | Response times slowed in Typ-AD and Amn-AD, not FTD |
| | Loss of track | Typ-AD present; FTD absent | Typ-AD lose track, request repetition of instructions |
| | Erratic, impulsive responses | FTD present; Typ-AD absent | FTD show erratic responding with poor use of feedback |

verbal digit, letter and word span tasks with phonological similarity and word length comparisons; pointing to body parts in order; visual pattern span test requiring memory of filled and unfilled squares on a grid); attention (sustained response to serial presentation of a single stimuli and a continuous performance task involving response to a designated stimuli among serially presented items); and executive function (a go:no-go inhibition task and a two-rule set-shifting task).

### 4. Results

Both typical-AD and FTD groups were impaired across tasks. Amnesic-AD patients performed well. Despite some similarities in overall score, typical-AD and FTD patients showed distinct performance profiles (Table 1). AD patients showed a profound deficit in repetition span, even on tasks without a dual-task component. They made omission errors and showed abnormal phonological similarity effects. FTD patients had difficulty on distraction tasks and made sequencing errors. Whereas FTD patients performed poorly on both sustained and continuous performance attentional tasks, AD patients were only impaired on the latter, the principal methodological difference being the amount of sequential information to be processed. On the go:no-go task typical-AD patients omitted both 'go' and 'no-go' targets, and asked for repetition of task instructions. The FTD group both omitted 'go' targets and made inhibition errors. On the set-shifting task both groups were impaired but typical-AD patients completed the task slowly and carefully. FTD patients responded quickly, and did not utilise feedback.

### 5. Discussion

Although both typical-AD and FTD groups were impaired across tasks, differences in profile suggest contrasting underlying deficits. 'Dysexecutive' features displayed in FTD (e.g. impaired inhibition, failure to use feedback, striking inattention) were absent in the AD group, who showed profound problems in working memory span and were consistently compromised when load was increased. These differences would be consistent with the notion that there are alternative, non-frontal contributions to the working memory problems in AD.

Verbal span deficits suggest a phonological contribution. Phonological impairments are reported by some authors (e.g. [5]), but there is a propensity to attribute such profiles to greater disease severity [6]. We would argue against this interpretation. Patients with severe span deficits are typically younger and demonstrate a distinct pattern of cortical symptoms. Illness duration and CDR scores were similar among all patient groups. We therefore propose that working memory problems are part of the posterior cortical symptomatology typical of youthful presentations of AD.

The typical-AD group also showed reduced visual span, suggesting that deficits cannot be entirely phonologically based. Span tasks were carried out in isolation, so deficits cannot be attributed to dual task impairment, as argued for other cohorts (e.g. [3,7]). Our data therefore suggest specific impairments in phonological and visuospatial short-term capacity and processing, rather than the central executive component of working memory. Notably, the deficit bears resemblance to that of 'short-term memory' patients (e.g. [8]), who, like this AD cohort, have lesions in temporoparietal cortex. Accumulating neuroimaging evidence further suggests a role for the posterior cortices in binding, retrieval, manipulation, and storage (e.g. [9]). The present study thus highlights the multiple contributions that may be involved in working memory deficits in AD, both pointing to a need for caution in the 'dysexecutive' interpretation of test failures and highlighting the potential role of the posterior hemispheres. They underline also the phenotypic variation within AD.

### References

[1] C.L. Stopford et al., Distinct memory profiles in Alzheimer's disease, *Cortex* **43** (2007), 846–857.

[2] J.S. Snowden et al., Cognitive phenotypes in Alzheimer's disease and genetic risk, *Cortex* **43** (2007), 835–845.

[3] A. Baddeley et al., Dementia and working memory, *Quarterly Journal of Experimental Psychology A* **38** (1986), 603–618.

[4] J.C. Thompson et al., Qualitative neuropsychological performance characteristics in frontotemporal dementia and Alzheimer's disease, *Journal of Neurology, Neurosurgery and Psychiatry* **76** (2005), 920–927.

[5] F. Collette et al., Phonological loop and central executive functioning in Alzheimer's disease, *Neuropsychologia* **37** (1999), 905–918.

[6] M.D. Kopelman, Rates of forgetting in Alzheimer-type dementia and Korsakoff's syndrome, *Neuropsychologia* **23** (1985), 623–638.

[7] R.G. Morris, Short term forgetting in senile dementia of the Alzheimer's type, *Cognitive Neuropsychology* **3** (1986), 77–97.

[8] E.M. Saffran and O.S. Marin, Immediate memory for word lists and sentences in a patient with deficient auditory short-term memory, *Brain and Language* **2** (1975), 420–433.

[9] K.L. Vilberg and M.D. Rugg, Memory retrieval and the parietal cortex: A review of evidence from a dual-process perspective, *Neuropsychologia* **46** (2008), 1787–1799.
